# Regular participation in leisure time activities and high cardiovascular fitness improve motor sequence learning in older adults

**DOI:** 10.1007/s00426-020-01351-y

**Published:** 2020-07-02

**Authors:** K. Zwingmann, L. Hübner, W. B. Verwey, J. S. Barnhoorn, B. Godde, C. Voelcker-Rehage

**Affiliations:** 1 Institute of Human Movement Science and Health, Chemnitz University of Technology, Chemnitz, Germany; 2 Cognitive Psychology and Ergonomics, University of Twente, Twente, The Netherlands; 3 Department of Health and Kinesiology, Texas A&M University, College Station, USA; 4 Human Behaviour & Organisational Innovation, The Netherlands Organization for Applied Scientific Research TNO, The Hague, The Netherlands; 5 Department of Psychology and Methods, Jacobs University Bremen, Bremen, Germany; 6 Institute of Sport and Exercise Sciences, University of Muenster, Muenster, Germany

## Abstract

**Introduction:**

Older adults show higher interindividual performance variability during the learning of new motor sequences than younger adults. It is largely unknown what factors contribute to this variability. This study aimed to, first, characterize age differences in motor sequence learning and, second, examine influencing factors for interindividual performance differences.

**Method:**

30 young adults (age *M* = 21.89,* SD* = 2.08, 20 female) and 29 older adults (age *M* = 69.55,* SD* = 3.03, 18 female) participated in the study. Motor sequence learning was assessed with a discrete sequence production (DSP) task, requiring key presses to a sequence of visual stimuli. Three DSP practice phases (á 8 blocks × 16 sequences, two six-element sequences) and two transfer blocks (new untrained sequences) were performed. Older participants conducted the Mini-Mental Status Examination and a visuospatial working-memory task. All participants finished a questionnaire on everyday leisure activities and a cardiovascular fitness test.

**Results:**

Performance speed increased with practice in both groups, but young improved more than older adults (significant Group × Time effect for response time, *F*(1,5) = 4.353, *p* = 0.004, $\eta_{p}^{2}$ = 0.071). Accuracy did not change in any age group (non-significant Group × Time effect for error rates, *F*(1,5) = 2.130, *p* = 0.091, $\eta_{p}^{2}$ = 0.036). Older adults revealed lower transfer costs for performance speed (significant Time × Group effect, e.g., simple sequence, *F*(1,2) = 10.511, *p* = 0.002, $\eta_{p}^{2}$ = 0.156). High participation in leisure time activities (*β* = − 0.58, *p* = 0.010, *R*^2^ = 0.45) and high cardiovascular fitness (*β* = − 0.49, *p* = 0.011, *R*^2^ = 0.45) predicted successful motor sequence learning in older adults.

**Discussion:**

Results confirmed impaired motor learning in older adults. Younger adults seem to show a better implicit knowledge of the practiced sequences compared to older adults. Regular participation in leisure time activities and cardiovascular fitness seem to prevent age-related decline and to facilitate motor sequence performance and motor sequence learning in older adults.

## Introduction

Learning new motor sequences is required for many everyday activities throughout the lifespan, like driving a car, operating a mobile phone and handling a medical device (Swanson & Lee, 1992). Older adults, too, need to learn or re-learn new motor sequences to maintain a self-determined daily living. Although the capability to learn new motor sequences persists in older adulthood (see King, Fogel, Albouy, & Doyon, 2013 for a review), older adults often learn more slowly than young adults and learning capacity increasingly varies with age (Daselaar, Rombouts, Veltman, Raaijmakers, & Jonkers, 2003; Hultsch, MacDonald, & Dixon, 2002; Wu & Hallet, 2005). Little is known about factors that determine this variability in learning rate of older adults. For example, lifestyle factors like the cardiovascular fitness level or leisure time activities as well as cognitive performance have been observed to influence motor performance and learning in persons of older age (Hübner & Voelcker-Rehage, 2017; Ren, Wu, Chan, & Yan, 2013). To contribute to the current research, we investigated potential factors that might explain interindividual differences in motor performance and sequence learning in older adults.

### Motor sequence learning in older adults

The Discrete Sequence Production (DSP) task is a commonly applied method to assess motor sequence learning. It requires responding to a sequence of visual stimuli by pressing the corresponding keys on a keyboard (Verwey, 1999). Another example of a motor sequencing task is the serial reaction time task, which, in contrast to the DSP task, implies no discrete sequences (Abrahamse, Ruitenberg, de Kleine, & Verwey, 2013). In general, older adults perform the DSP task more slowly than young adults (Barnhoorn, Döhring, Van Asseldonk, & Verwey, 2016; Barnhoorn, Van Asseldonk, & Verwey, 2017; Verwey, 2010). Still, older adults show considerable improvements in DSP task performance with practice, as reflected especially in an increased performance speed (Barnhoorn et al., 2016, 2017; Verwey, 2010). Even though it is widely accepted that performance improvement with practice is less in older than in young adults (Daselaar et al., 2003; Wu & Hallet, 2005), some studies reported little differences between young and older adults (Bhakuni & Mutha, 2015; Howard & Howard, 1989, 1992) or even steeper learning slopes in older adults (Brown, Robertson, & Press, 2009; Ehsani, Abdollahi, Mohseni Bandpei, Zahiri, & Jaberzadeh, 2015). Older adults might profit from faster acquisition of motor tasks in everyday life. In previous research, several factors have been explored as to their contribution to interindividual variability. These factors include developing explicit sequence knowledge (declines with age while implicit learning does not; King et al., 2013; Verneau, van der Kamp, Savelsbergh, & de Looze, 2014), complexity and duration of the task (age-related deficits with increased cognitive load and duration; King et al., 2013; Urry, Burns, & Baetu, 2018), and type of sequencing task (worse performance for repeated but not for random sequences; Shea, Park, & Braden, 2006). Age differences were also found with respect to different outcome parameters, i.e., speed or accuracy. For example, Verwey (2010) showed for the DSP task that learning rate was similar for young and older adults with respect to performance speed, but older adults had steeper learning slopes in terms of accuracy (i.e., percentage of errors). Further, measuring the transfer of sequence knowledge, i.e., when the nature of the movements is changed either to another sequence, to another part of the body or another measuring device, is one type of quantifying motor learning (Abrahamse et al., 2013). The better a task is learned, the higher might be the transfer costs to an unlearned task as the learned task is assumed to rely on integrated representations across task elements (Abrahamse, Jimenéz, Verwey, & Clegg, 2010). This involves the performance change caused by a transition from one sequence/body part/device to another. Transfer appeared less in older than in young adults in DSP tasks (lever vs. keypresses; Barnhoorn et al., 2016). This was attributed to older adults needing more time than young adults to develop implicit sequence knowledge (Verwey, 2001).

### Interindividual variability in motor sequence learning—factors associated with successful learning in older adults

Typically older adults show a higher interindividual variability in motor performance (Hultsch, MacDonald, & Dixon, 2002) and learning (Verwey, 2010; Voelcker-Rehage, 2008) than young adults. This variability has been attributed to several factors. One assumption is that a higher motor variability is caused by increased variability in neurobiological mechanisms (Myerson, Hale, Wagstaff, Poon, & Smith, 1990; Welford, 1980) such as variability in neurotransmitter functioning (Li & Lindenberger, 1999) and neural ‘noise’ in signal transmission (e.g., Hendrickson, 1982; Jensen, 1982; Li & Lindenberger, 1999). Also, differences in cognitive decline with aging have been argued to cause inter-individual variability in motor performance and learning in older adults. For example, Urry et al. (2018) revealed that learning differences in a serial reaction time task (a visuo-motor sequence learning task) were associated with general cognitive functions (fluid abilities). Bo and Seidler (2009) showed that visuospatial working-memory capacity predicted the rate of motor sequence learning in young adults. However, while general cognitive functions predicted performance level in older adults, it did not predict individual learning differences (Bo, Borza, & Seidler, 2009). Specifically, working-memory capacity and rate of motor learning in a color-cued explicit sequence task was reduced in older adults. Individual variations for the working-memory capacity were found, but did not correlate with the rate of motor learning. This suggests that other factors also influence motor learning (Bo et al., 2009).

Lifestyle factors such as cardiovascular fitness and (social) leisure time activities (e.g., painting or meeting with friends) might also cause interindividual variability in performance and learning capacity (and the reported performance differences) for the elderly. In older adults, cardiovascular fitness has been shown to correlate positively with brain function, brain structure and cognitive functioning (Voelcker-Rehage & Niemann, 2013). Moreover, cardiovascular fitness seems to be associated with the initial motor learning phase in complex upper extremity motor tasks, like visuospatial tracking tasks (see Hübner & Voelcker-Rehage, 2017 for an overview). With regard to leisure time activities, a systematic review by Stern and Munn (2010) revealed that participating in cognitive activities (e.g., reading, playing board games) may lead to a reduced risk of developing Alzheimer's disease and other dementias, and musical activity throughout the lifespan seems to help preserving cognitive functions in advanced age (Hanna-Pladdy & MacKay, 2011). As far as motor functions are concerned, in a longitudinal study lasting up to 11 years less participation in social activities was associated with a more rapid age-related decline in global motor functions (Buchman, Boyle, & Wilson, 2009). Finally, expertise studies indicate that work-related hand use (e.g., in precision mechanics) counteracts age-related decline in fine motor performance (Vieluf, Mahmoodi, Godde, Reuter, & Voelcker-Rehage, 2012; Vieluf et al., 2017). It must be considered that these findings are usually correlational and involve the problem that fit elderly may perform these activities more often. However, individual experiences and differences in daily use of the extremities should also be regarded when investigating individual differences in motor learning.

### The present study

The first aim of this study was to confirm the effect of age on motor sequence learning in the DSP task, quantified by performance speed and accuracy, in younger and older adults. Despite some heterogeneous results, based on the majority of findings in the current literature (e.g., Brown et al., 2009; Ehsani et al., 2015), we hypothesized that older adults would reveal poorer performance and slower motor learning as compared to young adults. More specifically, we expected that older adults would learn a simple motor sequence at a similar rate as young adults, but a complex sequence at a lower rate. Finally, as older adults are expected to automate a motor sequence more slowly and gain less explicit sequence knowledge than young adults (Verwey, 2010), we assumed that older adults would show less transfer costs during unfamiliar motor sequences.

The second aim of this study was to explore factors that are associated with successful learning and transfer in older adults that might explain interindividual variability in motor sequence learning. We hypothesized that performance gains due to practice would be positively correlated with cardiovascular fitness level (as indicated for other motor tasks; Hübner & Voelcker-Rehage, 2017), leisure time activities (Stern & Munn, 2010), daily hand use (analyzed exploratory) and visuospatial working memory capacity (Bo & Seidler, 2009, but see also Bo et al., 2009) in older participants. No profound hypotheses were derived for the correlation of these variables with motor learning *and* motor transfer and thus, they were analyzed on an exploratory level.

## Methods

### Participants

Thirty right-handed young adults (YA) between 20 and 30 years of age (21.89 ± 2.08, 20 female) and 30 right-handed older adults (OA) between 65 and 74 years of age (69.57 ± 2.99, 19 female) participated in the study. YA were recruited via personal contact, flyers, and student mailing lists. Participating students earned credits for their attendance. All OA participated in a previous study of our research group and had agreed to have their contact data stored in a participant database. They were re-recruited via phone and received 35 EUR for their participation. Exclusion criteria comprised motor impairment, neurologic diseases and history of cardiologic diseases. All participants completed a questionnaire assessing demographic information, health status, physical activity level (adapted version of Baecke, Burema, & Frijters, 1982), and handedness (Oldfield, 1971; only right handers were included). Manual dexterity was measured using the Purdue Pegboard test (model 32020, Lafayette Instruments, Lafayette, IN, USA; Tiffin & Asher, 1948) to rule out restrictions in fine motor performance. The mean number of pins out of three trials placed with the dominant right hand was calculated. All participants achieved a score within the normative values for the right hand (Desrosiers, Hébert, Bravo, & Dutil, 1995). After each practice session, participants were asked to rate the fatigue level of their right hand on a Visual Analog Scale from 0 to 10. This measure was included in the analysis to ensure that any age-related difference was not the result of fatigue, but of the experimental manipulation. No significant age-related differences in reported hand fatigue-level were found. OA were further screened for cognitive impairment with the Mini-Mental Status Examination (MMSE; cutoff < 27; Folstein, Folstein, & McHugh, 1975). One female OA was excluded because of limitations in using the fingers of the right hand. The remaining 29 OA (18 female) were between 65 and 74 years of age (69.55 ± 3.03, see Table 1). The study was approved by the Ethics Committee of the Faculty of Humanities of the Saarland University, Germany (4.3.13). The opportunity of quitting participation was granted and all participants were informed about the contents and goals of the study before signing consent. All OA provided consent from their personal physician for the cardiovascular fitness test.

**Table 1:** Participant characterization

	YA (*n* = 30, 20 female)		OA (*n* = 29, 18 female)		*F* statistics		
	*M*	*SD*	*M*	*SD*	*F*(1,59)	*p*	$\eta_{p}^{2}$
Age	21.89	2.08	69.55	3.03	4744.26	< 0.001**	0.988
Education	14.64	1.80	16.52	1.90	15.17	< 0.001**	0.210
Subj. health	4.37	0.62	4.03	0.63	4.23	0.044*	0.069
MMSE	–	–	28.45	0.97	–	–	–
Pegboard	15.90	1.31	12.41	1.56	86.19	< 0.001**	0.602
VO_2_-peak (l/min)	2.58	0.79	1.89	0.54	15.26	< 0.001**	0.211
MaxWatt/kg	3.07	0.41	2.00	0.52	76.34	< 0.001**	0.573

*YA* young adults, *OA* older adults, *Age* age in years, *Education* years of education, *Subj. health* self-rated health status in a Likert scale from 1 (poor) to 5 (excellent), *MMSE* sum score of the Mini Mental Status on Examination, *Pegboard* mean score of three trials with the right hand, *VO*_*2*_*-peak* peak oxygen consumption performed during cardiovascular fitness test, *MaxWatt/kg* maximal aerobic power

*p < 0.05, **p < 0.01

### Measures

#### Apparatus

The DSP task was programmed with E-Prime 2.0. Participants were seated in front of a 52 × 32.5 cm monitor (LCD monitor P2460PXQU, AOC International, Amsterdam, the Netherlands). The monitor was placed 65 cm from the edge of the table. Responses were given on a standard keyboard (Cherry DC 2000, ZF Friedrichshafen AG, Auerbach, Germany).

#### Discrete Sequence Production (DSP) task

Motor sequence learning was measured throughout 24 practice blocks and one transfer block of the DSP task (cf. below). As the rate of learning seems to depend on task complexity (King et al., 2013), sequences with two difficulty levels were performed. Participants were instructed to lay four fingers of their right hand, beginning with the index finger, on the keys C, V, B and N, respectively. Four white placeholders (3 cm width, 2.5 cm height) with black outlines were presented on a white screen, representing the four keys of the keyboard. When the white filling of one of the four placeholders on the screen turned green, the participant’s task was to react by pressing the corresponding key on the keyboard as fast and accurately as possible. After each response, the sequence continued with filling green one of the other placeholders. This ensured that the task was self-paced. Pressing a wrong key or not pressing a key within 2000 ms resulted in sequence abortion. Then the message “mistake” or “no reaction” (in German) appeared, and the next sequence started after 1000 ms. An entire sequence is denoted as a trial. The task instruction was presented on the screen at the beginning of each phase.

#### Sequences

The DSP task comprised two different sequences, consisting of six key presses each, which were constantly repeated in random order. One sequence consisted of a 2 × 3 order of the keys (the simple sequences included NCBNCB, VBCVBC, BNVBNV and CVNCVN; cf. Ruitenberg, Verwey, Schutter, & Abrahamse, 2014). The second sequence consisted of a 1 × 6 keying order (the complex sequences were BCVNVC, VNCBCN, NVBCBV and CBNVNB). Each participant always had one simple and one complex sequence, and these two always started with different first keys (e.g., NCBNCB and BCVNVC).

#### Blocks of the DSP task

To get familiar with the DSP task, participants started with a familiarization phase, during which one block of ten random three-key sequences and one block of ten random six-key-sequences were carried out. During practice, blocks 1–24 each comprised 16 sequences with each block including eight trials of simple and eight trials of complex sequences in random order. Participants were informed that they would practice two sequences. Each block was followed by a resting period of 20 s, during which a countdown was shown on the screen. In sum, every participant initiated 400 discrete sequences, involving 2400 key presses. Data collection of the DSP task ended with the transfer phase (block 25 + 26). It consisted of two blocks of 16 sequences with the practiced sequence and two blocks of 16 sequences with the newly generated, totally random and unfamiliar sequence. Half of the participants started with the unfamiliar sequence, the other half started with the practiced sequence. Participants were informed about the character (practiced or unfamiliar) of the sequences in advance.

#### Cardiovascular fitness test

To imply the cardiovascular fitness into the analysis as a possible factor for interindividual differences, participants completed a spiroergometry (ZAN600, nSpire Health, Oberthulba, Germany) on a bicycle ergometer (Lode Corival cpet, Groningen, the Netherlands). Peak oxygen consumption (VO_2_-peak) and maximal aerobic power (MaxWatt/kg) were assessed. This exertion test was designed as a ramp protocol (Hübner, Godde, & Voelcker-Rehage, 2018). Depending on the initial physical activity level of each participant, defined by the performance of more (equals the ‘fit’ protocol) or less (equals the ‘unfit’ protocol) than 3 h per week of endurance sports such as swimming, running and cycling, a particular Watt rate was chosen: The OA female ‘unfit’ protocol started with 10 W and contained a load of 10 W/min. The OA female ‘fit’, OA male ‘unfit’, and YA female ‘unfit’ protocol contained a load of 15 W/min, with 10 W as starting load. OA male ‘fit’, YA female ‘fit’ and YA male ‘unfit’ protocol contained a load of 20 W/min, with 20 W as starting load. The YA male ‘fit’ protocol started with 25 W and contained a load of 25 W/min. The protocols started with a 3-min rest period, duration of the load-phase varied between 8 and 12 min and after exertion participants finished with a 5-min cool-down-phase at a rate below 25 W. During the protocol the heart rate, an electrocardiogram (ECG, recorded with a ten-lead ECG fully digital stress system; Kiss, GE Healthcare, Munich, Germany) and blood pressure were recorded. In addition to that, different respiratory parameters were registered, e.g., the level of oxygen and carbon dioxide or the number of breaths per minute. Participants were told to keep their revolutions per minute (rpm) between 60 and 80 and to cycle until their subjective exertion. Objective exertion criteria for OA consisted of a respiratory quotient (ratio of carbon dioxide to oxygen) of 1.1 for about 30 s (YA: 1.3 for 30 s), a blood pressure above 230/115 mmHg, or a heart rate above 220 minus the age of the respective participant. Cardiovascular fitness tests were supervised by an experienced sports scientist. The Watt values of the highest completed performance level were averaged and regarded as cardiovascular fitness, expressed as maximal aerobic power. VO_2_-peak was calculated by the mean of the last five values of the highest completed performance level.

#### Visuospatial working-memory task

A modified version of the visuospatial working-memory task used also by Bo et al. (2009; original task by Luck and Vogel 1997) was utilized to assess visuospatial working-memory in the older adults (i.e., we reduced the number of array sizes from 10 to eight, lowered the amount of experimental trials, and participants determined the start of the upcoming trial by pressing the space bar). Keyboard and monitor setup was identical to the DSP task. During the visuospatial working-memory task, 2, 4, 6 or 8 squares (size of 0.7 cm × 0.7 cm) were presented for 100 ms in seven different colors (black, white, blue, red, green, yellow, and violet). This was followed by a 900 ms blank screen delay which was followed by presenting the same number of squares, now with one square encircled in red for 2000 ms. It was the participant’s task to decide whether or not the encircled square had changed color by pressing the appropriate key (“A” for unchanged, “L” for changed). Participants received visual feedback regarding their decision after each trial (“correct” or “wrong” displayed in German). In case no response was given within 2000 ms, the message “unfortunately too slow” appeared. After ten practice trials, participants continued with 120 experimental trials. A memory capacity score was calculated in accordance to Bo et al. (2009): *K* = *S* (*H* − *F*); *S* = size of the array, *H* = observed hit rate, *F* = false alarm rate. The average *K* (across all array sizes) was used to quantify the visuospatial working-memory capacity. Young adults did not perform the visuospatial working-memory task.

#### Leisure time activities

A questionnaire adapted from the German Socio-Economic Panel (SOEP; Wagner, Frick, & Schupp, 2007 for more information) and others (Aartsen, Smits, van Tilburg, Knipscheer, & Deeg, 2002; Hultsch, Hammer, & Small, 1993; Maier & Klumb, 2002) was used to determine leisure time activities (cf. Niemann, Godde, Staudinger, & Voelcker-Rehage, 2014). Participation in 17 everyday activities (i.e., visiting cultural events, meeting with friends, performing creative activities, voluntary or care services, gambling or working on puzzles, further education, sports, religious activities, shopping, household chores, gardening, listening to music, watching TV, reading and computer work) were assessed on a five-point Likert scale between never/less than once per month and several times a week. The mean out of the 17 items served as the leisure time activity score.

#### Hand use

Daily hand use was assessed with a questionnaire asking how often the participant performs the following manual activities (six-point Likert scale from never to very often): playing a musical instrument, using computer keyboard/typewriter, writing by hand, needlework (e.g., knitting), model building/tinker, other fine motor skills (Vieluf et al., 2012). The sum out of the six items represented the score for daily hand use.

### Procedure

**Fig. 1:**
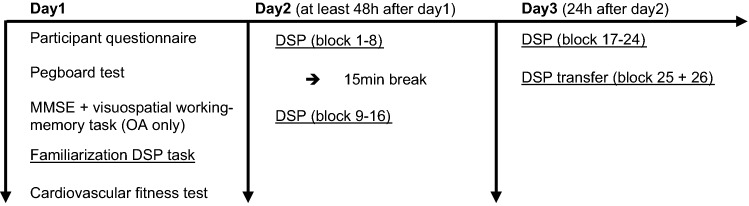
Study procedure. *DSP* Discrete Sequence Production, *MMSE* Mini-Mental Status Examination, *OA* older adults (created with Word by Microsoft Office Professional Plus 2013)

Figure 1 illustrates the experimental procedure. Participants came to the lab on three different testing days. The Day 1 session started with providing general information and giving informed consent about the project after which the questionnaire was filled out. Both groups performed the Purdue Pegboard Test (Tiffin & Asher, 1948). OA additionally performed the MMSE and visuospatial working-memory task. Afterwards, the familiarization phase of the DSP task for both groups and the cardiovascular fitness test were performed. Altogether, the session on the first day lasted about 90 min.

Day 2 session: To ensure full recovery from the cardiovascular fitness tests, day 2 started at least 48 h after day 1. Before starting with the DSP task participants were told that the task involves two sequences consisting of six keypresses each and that these sequences would be repeated in a random order. Participants were not informed about the different complexities of the two sequences. The DSP task started with block 1–8. Block 9–16 were carried out 15 min after the end of block 8. The procedures on the second day took on average 45 min.

Day 3 session: 24 h after the beginning of block 1, participants executed block 17–26 of the DSP task. The procedures on the third day took on average 30 min.

### Data analysis

Performance speed was quantified by the response time (RT) in ms, defined as the elapsed time from the presentation of a visual stimulus to the correct keypress on the keyboard. Accuracy was quantified by the error rate (ER). An error trial was defined as a trial during which an error was committed. ER was computed by calculating a percentage value of all error trials in relation to all executed trials (a high ER implies a low accuracy). The familiarization phase as well as the familiar block of the transfer phase was not analyzed. Error trials, outliers as well as the first trial of each block were excluded from RT data analyses. Outlier values were defined as 2.5 times the standard deviation above the mean performance speed of each block. A learning rate (LR) index was composed by subtracting the mean RT of block 24 from the first block, separately for every participant of OA and YA as well as for the simple and complex sequence.

### Statistical analysis

**Fig. 2:**
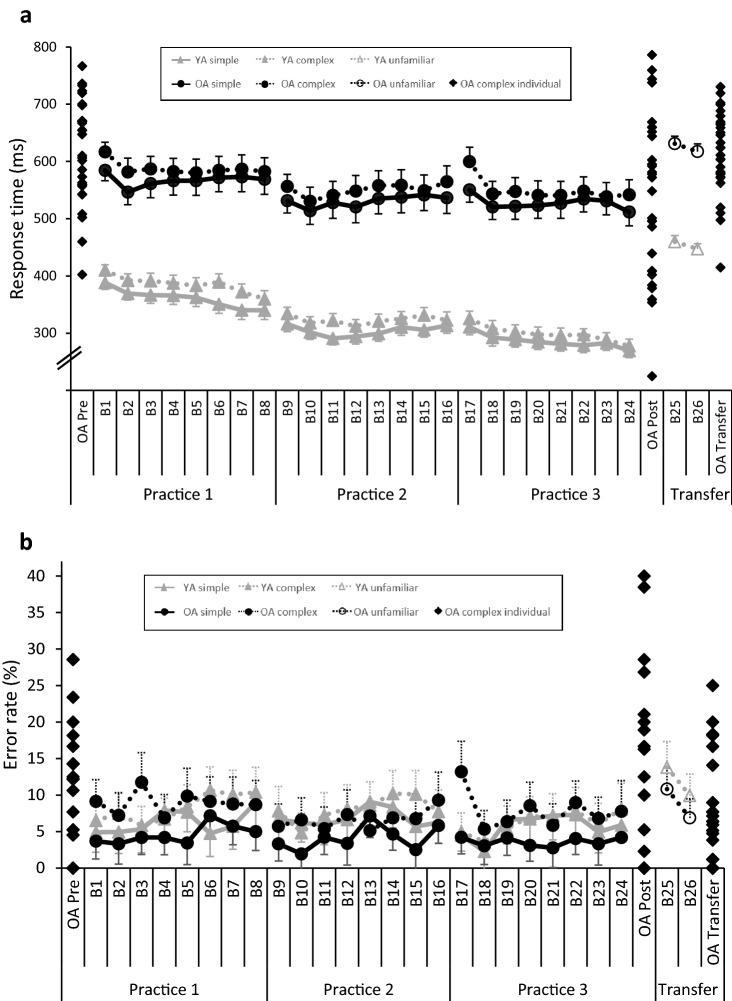
**a** Development of response times (RT) of older adults (OA) and younger adults (YA) throughout DSP practice on day 2 and DSP practice and transfer phase of the unfamiliar sequence on day 3. The variable is displayed as mean with standard error. For the complex sequence of the OA, the individual starting level (OA Pre, equates to block 1), individual end level (OA Post, equates to block 24) and individual transfer level (OA transfer, equates to block 26) are displayed (created with Excel by Microsoft Office Professional Plus 2013). **b** Development of error rates (ER) of older adults (OA) and younger adults (YA) throughout DSP practice on day 2 and DSP practice and transfer phase of the unfamiliar sequence on day 3. The variable is displayed as mean with standard error. For the complex sequence of the OA, the individual starting level (OA Pre, equates to block 1), individual end level (OA Post, equates to block 24) and individual transfer level (OA transfer, equates to block 26) are displayed (created with Excel by Microsoft Office Professional Plus 2013)

To investigate age effects on motor sequence learning, we conducted separate 6 Time (block 1, block 8, block 9, block 16, block 17, block 24) × 2 Sequence (complex, simple) × 2 Group (OA, YA) repeated measures ANOVAs (for block classification see Fig. 2) for mean RT and mean ER. Bonferroni corrected post-hoc tests (pairwise comparisons) were performed to detect the difference between the single points of Time, the Sequences and Group. Transfer of sequence knowledge was analyzed by 2 Time (block 24, block 25/26) × 2 Group (OA, YA) repeated measures ANOVAs for RT and ER (one ANOVA for the transition between practice *simple* [block 24] to transfer [block 25/26] and one ANOVA for the transition between practice *complex* [block 24] to transfer [block 25/26]). Note that either block 25 or 26 (randomized) only contained unfamiliar sequences. For all ANOVAs Greenhouse–Geisser adjustment was used in case the sphericity assumption was violated. Effect sizes are reported as partial eta squared ($\eta_{p}^{2}$).

To examine factors that might determine the learning rate for the complex sequence (RT block 24 complex—RT block 1 complex) and transfer (RT block 25/26 random—RT block 24 complex), Pearson correlations were computed for age, years of education, visuospatial working-memory task capacity score (OA only), maximal aerobic power (further referred to as cardiovascular fitness level), leisure time activities, and hand use. Correlation analyses were performed with the whole study sample as well as for each age group (YA, OA) separately. In a second step, variables revealing a significant association with at least one of the depended variables (learning, transfer) in the analyses of the whole sample or the single age group samples were included in two sets of stepwise multiple regression models using the backward method aiming to predict (a) learning and (b) transfer. In the final regression analyses, hand use, leisure time activities and cardiovascular fitness were used as predictors for the group of OA only. In all statistical analyses *p* values ≤ 0.05 were regarded as significant and *p* values ≤ 0.10 as marginally significant.

## Results

### Age-differences in motor learning

#### Performance speed

Overall, RTs of OA were longer than of YA. The variability between OA was higher than between YA, indicated by higher standard errors. For both groups, RTs for the simple sequence were significantly shorter than for the complex sequence (cf. Tables 2, 3). Figure 2a illustrates that irrespective of Group and Sequence, RT decreased with practice and for both groups RT of the simple sequence decreased in parallel to RT of the complex sequence.

**Table 2:** Mean response time (RT) and mean error rate (ER) for young (YA) and older adults (OA) per sequence type (simple and complex: both mean of block 1–block 24, unfamiliar: mean of block 25–block 26)

	YA						OA					
	Simple		Complex		Unfamiliar		Simple		Complex		Unfamiliar	
	*M*	*SD*	*M*	*SD*	*M*	*SD*	*M*	*SD*	*M*	*SD*	*M*	*SD*
RT (ms)	316.79	13.53	336.07	12.68	453.47	9.98	541.77	24.44	562.84	24.36	624.26	13.13
ER (%)	6.29	3.29	7.54	3.02	11.87	3.26	4.12	2.58	7.82	3.29	8.85	2.77

**Table 3:** *F* statistics for the 6 Time (block 1, block 8, block 9, block 16, block 17, block 24) × 2 Sequence (simple, complex) × 2 Group (older adults, young adults) ANOVAs with repeated measures for response time (RT; in ms) and accuracy (ER; in %)

	Response time				Error rate			
	*df*	*F*	*p*	$\eta_{p}^{2}$	*df*	*F*	*p*	$\eta_{p}^{2}$
Time	5	43.431	< 0.001**	0.432	5	1.344	0.260	0.023
Group	1	92.977	< 0.001**	0.960	1	0.008	0.927	< 0.001
Sequence	1	17.202	< 0.001**	0.232	1	15.387	< 0.001**	0.213
Time × Group	5	4.353	0.004**	0.071	5	2.130	0.091	0.036
Group × Sequence	1	1.724	0.194	0.029	1	5.736	0.020*	0.091
Time × Sequence	5	1.354	0.246	0.023	5	0.808	0.500	0.014
Group × Time × Sequence	5	1.969	0.091	0.033	5	0.854	0.474	0.015

*p < 0.05, **p < 0.01

These observations were confirmed by a 6 Time (block 8, block 9, block 16, block 17, block 24) × 2 Sequence (complex, simple) × 2 Group (OA, YA) repeated measures ANOVA indicating significant main effects of Time, Group and Sequence, but no significant Time × Sequence interaction (cf. Table 3). A significant Group × Time interaction revealed less performance increase for OA than for YA over time. For OA, for each sequence, RT decreased significantly from the first to the last practice block and was always significantly faster than in the first block of practice (block 1 to block 9, block 1 to block 16, block 1 to block 24, block 17 to block 24). Also for YA, RT decreased significantly with practice (exception: block 9 to block 16). Concerning learning rate, the relationship between sequence type and time tends to be different for the age groups (Table 3). Comparing the RT between the complex and the simple sequence at the different points of time, the OA revealed faster RT in the simple than in the complex sequences (all except block 8). In contrast, for the YA only in block 1 and block 9 the simplex sequence was performed significantly faster than the complex sequence. This illustrates a distinct difference between the two sequences for the OA, but not for the YA.

#### Accuracy

Figure 2b shows that accuracy, quantified by the error rate (ER), did not decrease with practice, but remained relatively stable. Moreover, no clear difference between YA and OA is visible. This was confirmed by a 6 Time (block 1, block 8, block 9, block 16, block 17, block 24) × 2 Sequence (complex, simple) × 2 Group (OA, YA) repeated measures ANOVA, indicating no main effect of Time or Group. A significant effect of Sequence indicated overall higher ER for the complex than for the simple sequence, which was mainly driven by a high difference in OA (Group × Sequence interaction, cf. also Table 2). A marginally significant Group × Time interaction suggests different development over time: ER of YA increased from block 1 to block 8 and slightly decreased afterwards, whereas ER of OA remained relatively stable (only ER in block 9 was lower as compared to the other blocks). Bonferroni corrected post-hoc pairwise comparisons did not detect any significant differences for ER between the measurement time points for both groups and both sequences. Only for the OA, the post-hoc comparisons revealed for block 1, block 17 and block 24 a difference in ER.

### Age-differences in motor transfer

#### Performance speed

Motor transfer was analyzed by comparing RT of the last practice block (block 24) with the first block of the transfer phase (block 25/26). Again, YA performed faster than OA. Both groups revealed longer RTs during the unfamiliar sequence at block 25/26 as compared to the practiced sequence at block 24 (cf. Fig. 2). This was confirmed by the main effects of Group and Time in 2 Time × 2 Group repeated measures ANOVAs for the simple as well as the complex sequence (cf. Table 4). YA demonstrated a higher increase in RT from block 24 to block 25/26 than OA as indicated by Time × Group interactions.

**Table 4:** *F* statistics for the 2 Time (block 24, block 25) × 2 Group (older adults, young adults) ANOVAs with repeated measures for response time (RT; in ms) and accuracy (ER; in %) of the transfer of learning

		Simple				Complex		
	Response time							
	*df*	*F*	*p*	$\eta_{p}^{2}$	*df*	*F*	*p*	$\eta_{p}^{2}$
Time	1	200.029	< 0.001**	0.778	1	135.494	< 0.001**	0.704
Group	1	113.714	< 0.001**	0.666	1	116.121	< 0.001**	0.671
Time × Group	1	10.511	0.002**	0.156	1	15.830	< 0.001**	0.217

	Error rate							
Time	1	21.614	< 0.001**	0.275	1	0.656	0.421	0.011
Group	1	74.740	0.249	0.023	1	0.134	0.716	0.002
Time × Group	1	0.175	0.678	0.003	1	3.841	0.055	0.063

*p < 0.05, **p < 0.01

#### Accuracy

As already indicated for the practice block 1–24, YA and OA did also not show changes in ER during block 24 and block 25/26 (no significant main effects of Group of the 2 Time × 2 Group repeated measures ANOVAs). For the simple sequence, both groups had a higher ER during unfamiliar sequence in block 25/26 compared to the practiced sequence in block 24 (main effect of Time, but no Time × Group interaction). For the complex sequence, a marginal significant Time × Group interaction indicated that the ER of YA increased from block 24 to block 25/26 whereas ER of OA remained stable. Overall, OA showed less performance decline in terms of speed and accuracy than YA during block 25/26 of the transfer phase.

### Factors that might determine motor learning and transfer

Correlation analyses were performed for variables that might influence the learning rate (block 24–block 1) and motor transfer (block 25/26–block 24; see Tables 5, 6, 7). Irrespective of the analyzed sample (OA and YA collapsed or YA and OA alone), a high amount of learning was associated with high transfer costs. Furthermore, learning was positively associated with leisure time activities. For YA, neither learning nor transfer correlated significantly with cardiovascular fitness level, leisure time activities, or hand use (see Table 6). For OA, learning was positively associated with leisure time activities and daily hand use (see Table 7). Transfer was negatively associated with leisure time activities and marginally significant with daily hand use (*r* = 0.359, *p* = 0.056; see Table 7). Age, years of education, and capacity score of the visuospatial working-memory task revealed no significant associations with learning or transfer for OA. Thus, they were not included in the ensuing regression analyses. Due to a significant correlation with learning and transfer in the whole sample, but not in OA, cardiovascular fitness was considered in the regression model. Taken together, results indicated that correlation analyses of the whole study sample were driven by the results of OA. Therefore, regression analyses were conducted with leisure time activities, daily hand use and cardiovascular fitness as predictors for the group of OA only.

**Table 5:** Intercorrelations for learning and transfer with age, years of education, visuospatial working-memory task, cardiovascular fitness, leisure time activities and hand use for all participants (*n* = 59)

Measure	1a	1b	2	3	4	5	6
1a. Learning	–						
1b. Transfer	− 0.811**	–					
2. Age	0.351**	− 0.457**	–				
3. Education	0.003	− 0.019	0.481**	–			
4. Visuospatial working-memory task	–	–	–	–	–	–	–
5. Cardiovascular fitness level	− 0.322*	− 0.357*	− 0.744**	− 0.316*	–	–	
6. Leisure time activities	− 0.283*	0.186	0.198	0.316*	–	− 0.325*	–
7. Hand use	− 0.244	0.202	0.102	0.154	–	− 0.181	0.460**

*p < 0.05, **p < 0.01

**Table 6:** Intercorrelations for learning and transfer with age, years of education, visuospatial working-memory task, cardiovascular fitness, leisure time activities and hand use for young adults (*n* = 30)

Measure	1a	1b	2	3	4	5	6
1a. Learning	–						
1b. Transfer	0.685**	–					
2. Age	− 0.107	0.130	–				
3. Education	− 0.059	0.027	0.675**	–			
4. Visuospatial working-memory task	–	–	–	–	–		
5. Cardiovascular fitness level	− 0.110	− 0.073	0.304	0.187	–	–	
6. Leisure time activities	− 0.223	0.105	− 0.083	0.015	–	− 0.542	–
7. Hand use	− 0.004	0.168	0.039	− 0.122	–	− 0.317	0.235

*p < 0.05, **p < 0.01

**Table 7:** Intercorrelations for learning and transfer with age, years of education, visuospatial working-memory task, cardiovascular fitness, leisure time activities and hand use for older adults (*n* = 29)

Measure	1a	1b	2	3	4	5	6
1a. Learning	–						
1b. Transfer	− 0.813**	–					
2. Age	0.093	0.051	–				
3. Education	− 0.269	0.359	− 0.017	–			
4. Visuospatial working-memory task	− 0.119	0.162	− 0.374*	0.078	–		
5. Cardiovascular fitness level	− 0.090	0.033	0.012	− 0.052	0.344	–	
6. Leisure time activities	− 0.477**	0.424*	− 0.084	0.451*	− 0.102	− 0.542**	–
7. Hand use	− 0.462**	0.359	− 0.312	0.303	− 0.001	− 0.317	0.600**

*p < 0.05, **p < 0.01

#### Learning

The variables hand use, leisure time activities, and cardiovascular fitness were entered in the stepwise multiple regression analysis aiming to predict motor learning in OA (see Table 8 for statistical results). Model 1 (*F*(1,28) = 6.69, *p* = 0.002) had the highest explained variance of 45%. The variables leisure time activities (*p* = 0.010) and cardiovascular fitness (*p* = 0.011) were significant, indicating that higher leisure time activities and higher cardiovascular fitness were associated with better motor learning in OA.

**Table 8:** Stepwise regression analysis aiming to predict (a) learning and (b) transfer for older adults

(a) Learning	Model			
	1		2	
	*B*	*β*	*B*	*β*
Hand use	− 5.00	− 0.27		
Leisure time activities	− 110.90	− 0.58*	− 141.87	− 0.74**
Cardiovascular fitness	− 91.59	− 0.49*	− 92.19	− 0.49*
Total *R*^2^	0.45*		0.40	
Total Δ*R*^2^			− 0.05	

(b) Transfer	Model			
	1		2	
	*B*	*β*	*B*	*β*
Hand use	3.59	0.16		
Leisure time activities	123.48	0.53*	145.73	0.63*
Cardiovascular fitness	84.59	0.37	85.02	0.37
Total *R*^2^	0.29*		0.28	
Total Δ*R*^2^			− 0.02	

*p < 0.05, **p < 0.01

#### Transfer

In the second stepwise multiple regression analyses, the variables hand use, leisure time activities, and cardiovascular fitness were expected to predict motor transfer in OA (see Table 8 for statistical results). Again, model 1 (*F*(1,28) = 3.47, *p* = 0.031), considering all three variables had the highest explained variance of 29%. Leisure time activities (*p* = 0.034) and cardiovascular fitness (*p* = 0.076) revealed a (marginal) significant effect. High transfer costs from the practiced sequence to the unfamiliar sequence (i.e., high difference in performance speed, interpreted as a sign of high internalization), was associated with high participation in leisure time activities and high cardiovascular fitness.

## Discussion

We investigated the influence of age on motor sequence learning in the Discrete Sequence Production (DSP) task with a complex and a simple sequence. In summary, the young outperformed the older adults with overall faster performance speed (quantified by response time) irrespective of sequence, but no age difference was found with respect to accuracy (quantified by the error rate). In both groups, performance speed increased with practice, but the young improved more than the older adults. In contrast, accuracy did not change significantly with practice for any age group. Older adults showed higher transfer in terms of performance speed than young adults. High participation in leisure time activities and high cardiovascular fitness predicted successful learning and higher transfer costs in older adults.

### Age-differences in motor sequence learning

Although older adults increased their performance speed significantly with practice, they showed less improvement than young adults, confirming our hypotheses. Additionally, both age groups revealed faster performance speed during the execution of the simple as compared to the complex sequence. Reported results corroborate previous evidence that aspects of motor learning are impaired in older adults (Daselaar et al., 2003; Wu & Hallet, 2005) and thus contradict findings showing no age differences in motor learning (Bhakuni and Mutha, 2015; Brown et al., 2009; Ehsani et al., 2015; Howard & Howard, 1989, 1992; Verwey, 2010 for initial practice). The decrement in learning plasticity might be explained by several age-related physiological and neurophysiological changes (for overviews see Seidler et al., 2010; Voelcker-Rehage, 2008), but might also be specific to the study design (cf. Verwey, 2010).

In contrast to performance speed, accuracy did not change significantly with practice. Although the analyses revealed a marginally significant Time by Group interaction, no clear improvement or deterioration was detected in young nor in older adults. Both groups developed differently during practice with older adults showing less performance increase than young adults over time. This is in line with earlier studies using the DSP task (Barnhoorn et al., 2016, 2017). Only in older adults error rate was higher with the complex than with the simple sequence. Young adults showed no effect of sequence complexity on error rate. Older adults did not only improve their performance speed less than young adults in the complex but also in the simple sequence. These results underline the high difficulty level of DSP task performance for older adults, especially for the complex sequence.

Noteworthy, older adults also demonstrated much higher performance variability than young adults as indicated by (descriptively) higher standard errors for speed and accuracy, visible at the individual start, end and transfer levels as revealed in Fig. 2a, b. A temporary lapse of attention (Bunce, Warr, & Cochrane, 1993) or reduced executive control (West, Murphy, Armilio, Craik, & Stuss, 2002) might be reasons for the greater performance variability in older adults. Other reasons for this phenomenon may be external (e.g., motivation) or strategic (adjustment from the old to a new learning strategy). Additionally, limitations of the performance measures are conceivable, i.e., either the DSP task being too easy (ceiling effects) or too difficult (floor effects) for some of the older adults. We do not assume to have ceiling or floor effects since our plotted individual data in Fig. 2a, b do not show such. As the subjective fatigue did not change with practice and did not differ between age groups, fatigue can be excluded as an influencing factor.

### Age-differences in transfer costs

Consistent with our hypotheses, older adults showed less transfer costs compared to young adults with respect to performance speed, indicated by less speed reduction from the practiced to the unfamiliar motor sequences. Considering the following interpretation, less transfer costs imply an inferior transfer. Bock (2005) suggested that adaptation-related cognitive resources might decline in old age, making it more difficult for seniors to improve performance and automate a task. Lower performance improvements combined with the lower transfer of sequencing skill for both used sequences in older adults, thus, might be a sign of less automatization. The higher transfer cost in young adults on the contrary might be interpreted in the way that young compared to older adults might have already better internalized the practiced sequences (Abrahamse et al., 2013; Barnhoorn et al., 2016; Verwey, 2001), and consequently had higher transfer costs. Thus, young adults seem to engage in more *sequence-specific* learning while older adults learn more *task-general* (Verwey, 2010). This interpretation was supported by significant correlations between the learning rate and factors that might influence it (e.g., age, cardiovascular fitness level, leisure time activities). Additionally, according to Seidler (2010), for learning and transfer different brain regions are required. Contrary to the learning region, the transfer region seems to be affected by the process of aging. It remains speculative whether transfer costs would have been higher in older adults with longer practice.

### Factors that are associated with motor sequence learning and transfer in older adults

Despite a non-significant association between cardiovascular fitness and motor learning, cardiovascular fitness was added in the regression analysis to exclude the possibility that influence of leisure time activities on motor learning is caused by more fit older adults also performing more leisure time activities, but rather to separate these variables. Consequently, high participation in leisure time activities and high cardiovascular fitness were associated with successful learning (i.e., a high-performance gain from the first to the last block of practice) in the DSP task in older adults. This finding is in line with results from several studies revealing a positive association of participating in leisure time activities and preserved cognitive (Stern & Munn, 2010; Verghese et al., 2003) as well as motor functions (Buchman et al., 2009) in older adults. Although the exact mechanisms remain unknown, Buchman et al. (2009) suggested that leisure time activities and motor function might require overlapping neural systems. Thus, to activate resources and support general motor functions in older adults, certain leisure time activities can be recommended. According to the interpretation of Buchman et al. (2009), regular participation in leisure time activities might trigger neural plasticity in the joint elements of the neural system, leading to higher levels of learning. As leisure time activity also has been shown to be associated with a reduced risk of Alzheimer’s disease, one might assume that it contributes to maintaining brain health and/or to prevent structural and functional brain decline (Stern & Munn, 2010). Furthermore, a high cardiovascular fitness level seems to promote motor sequence learning in the DSP task in older adults. This effect seems to be mediated by molecular, cellular and functional changes in the brain induced by regular exercise (El-Sayes, Harasym, Turco, Locke, & Nelson, 2018; Lehmann & Taubert, 2018 for details).

We know, that motor learning has been related to cognitive performance, e.g., working memory (Bo, Borza, & Seidler, 2009) and that the fitness level has been associated with executive functions (Colcombe & Kramer, 2003). Therefore, it might be possible that the effect of cardiovascular fitness and leisure time activities on motor learning is mediated by cognitive performance. Unfortunately, with the cognitive task used in our study design (visuospatial working-memory task), we were not able to replicate this effect within the sample of older adults and we did not assess this measure for young adults (for a more detailed discussion with respect to a fine motor task see Hübner, Vieluf, Godde, & Voelcker-Rehage, 2019). The second regression analysis revealed that participation in leisure time activities and cardiovascular fitness (marginal significant) further predicted a high-performance decline during the transfer phase in older adults. Performance decline was reflected by a high gain in reaction times when switching from practiced sequence to unfamiliar sequence in the transfer phase and represent a performance decrease. As argued above for the old-young comparison, high transfer costs seem to indicate that participants have automated the practiced sequences and that this internalization impedes fast execution of unfamiliar sequences. The association of a high participation rate in leisure time activities and cardiovascular fitness with higher transfer costs in older adults was further supported by a significant medium–high positive correlation between leisure time activities and transfer (*r* = 0.424, *p* = 0.022). Accordingly, our data might indicate that older adults participating a lot in leisure time activities and with a higher cardiovascular fitness level might also have internalized the sequences better than older adults with a lower participation in leisure time activities and low cardiovascular fitness. Therefore, participation in leisure time activities and cardiovascular fitness in older adulthood seems to not only facilitate a higher learning rate, but also a better internalization of learned sequences.

None of the other investigated variables (age, education, visuospatial working-memory) were associated with learning or transfer. Analyses revealed that visuospatial working-memory capacity does not predict the rate of motor sequence learning in older adults (Bo et al., 2009), although this effect was found in young adults (Bo & Seidler, 2009). Bo et al. (2009) suggested that the missing association in older adults might be due to the high interindividual learning differences in this age group.

## Limitations

Despite the recommendation by Abrahamse et al. (2013) concerning the repetitions of sequences during learning (500–1000 repetitions) we only used 400 repetitions of the sequences in our study. More extensive learning sessions, however, would have led to fatigue (Barnhoorn et al., 2017) which in turn might have impacted the results for both age groups. Therefore, we distributed the learning sessions on two consecutive days instead of one long session. Consequently, the present results hold for moderate but not for extensive practice.

Different leisure time activities with the hands can correlate with each other, e.g., video gaming and piano playing (for young adults; Verwey et al., 2015; Verwey & Wright, 2014). We used validated scales to determine leisure time activities (Niemann et al., 2014) and hand use (Vieluf et al., 2012). However, the leisure time activity scale incorporated three (out of 17) items that assessed activities performed with the hands, i.e., (1) working on puzzles, (2) playing games, and (3) using internet/computer). This overlap was reflected by significant correlations between leisure time activities and hand use for the whole sample (*r* = 0.460, *p* < 0.001) and older adults (*r* = 0.600, *p* = 0.001), but not young adults (*r* = 0.235, *p* = 0.212). Therefore, including leisure time activities into the regression analysis might take significance from the factor hand use. Nevertheless, we decided to perform analyses with the whole approved scales. Additionally, we decided to add the cardiovascular fitness level into subsequent regression analysis as we detected a significant correlation with motor learning and transfer for the whole sample (although this was a non-significant correlation in the single age groups), indicating a possible influence on motor sequence learning. In order to identify all possible influencing parameters, we added this variable into regression analysis.

For cardiovascular assessment, it is suggested to adapt the work rate in a way that the participants reach their performance limits in about 10 min (see e.g., Buchfuhrer, Hansen, Robinson, Sue, Wasserman, & Whipp, 1983). To account for sex and different levels of cardiovascular fitness in our sample, the test protocol, therefore, was adapted based on participants self-reported fitness status (cf. Hübner et al., 2018). We cannot preclude that protocol selection may have an influence on submaximal and maximal physiological outcomes, such as maximal aerobic power (Bentley, Newell, & Bishop, 2007) or VO_2_-peak (Roone & Bourgois, 2012), and that the sensitivity of the fitness parameter may be reduced by this factor. However, protocol runtime should be based upon the expected capacity of the examined person to avoid influences of fatigue (too long protocol) or a medium load (too short protocol; Breuer, 2004).

## Conclusions

We aimed to examine the effects of age on motor sequence learning and to explain interindividual learning differences in older adults. Younger adults showed overall a faster performance speed than older adults, but age groups did not differ with respect to accuracy. Further, older adults showed less transfer costs when switching from practiced to unfamiliar sequences, which was interpreted as indicating that they internalized the sequence less than young adults. The huge interindividual variability in learning, particularly in older adults, advises aging research and clinical practice to personalize interventions and/or use case-by-case reviews instead of mean values or standardized programs.

This study further suggests that leisure time activities and cardiovascular fitness positively predicted motor sequence learning in older adults. Moreover, high levels of leisure time activities and cardiovascular fitness predicted high transfer costs which might indicate that highly active older adults internalized the sequences more than less active older adults. Thus, this study confirms social engagement and cardiovascular fitness as factors for successful aging so that we can conclude that being active supports motor sequence learning in older adulthood.

## Data Availability

The authors allow the journal to review the datasets if requested. The authors have full control of all primary datasets and analyses during the current study and these datasets are available from the corresponding author on reasonable request.
