# Correction to: Regular participation in leisure time activities and high cardiovascular fitness improve motor sequence learning in older adults

**DOI:** 10.1007/s00426-021-01558-7

**Published:** 2021-07-18

**Authors:** K. Zwingmann, L. Hübner, W. B. Verwey, J. S. Barnhoorn, B. Godde, C. Voelcker-Rehage

**Affiliations:** 1 Institute of Human Movement Science and Health, Chemnitz University of Technology, Chemnitz, Germany; 2 Cognitive Psychology and Ergonomics, University of Twente, Twente, The Netherlands; 3 Department of Health and Kinesiology, Texas A&M University, College Station, USA; 4 Human Behaviour & Organisational Innovation, The Netherlands Organization for Applied Scientific Research TNO, The Hague, The Netherlands; 5 Department of Psychology and Methods, Jacobs University Bremen, Bremen, Germany; 6 Institute of Sport and Exercise Sciences, University of Muenster, Muenster, Germany

## Correction to: Psychological Research (2021) 85:1488–1502 10.1007/s00426-020-01351-y

The article “Regular participation in leisure time activities and high cardiovascular fitness improve motor sequence learning in older adults”, written by K. Zwingmann, L. Hübner, W. B. Verwey, J. S. Barnhoorn, B. Godde and C. Voelcker-Rehage, was originally published Online First without Open Access. After publication in volume 85, issue 4, pages 1488–1502 the author decided to opt for Open Choice and to make the article an Open Access publication. Therefore, the copyright of the article has been changed to © The Author(s) 2020 and the article is forthwith distributed under the terms of the Creative Commons Attribution 4.0 International License, which permits use, sharing, adaptation, distribution and reproduction in any medium or format, as long as you give appropriate credit to the original author(s) and the source, provide a link to the Creative Commons licence, and indicate if changes were made. The images or other third party material in this article are included in the article’s Creative Commons licence, unless indicated otherwise in a credit line to the material. If material is not included in the article’s Creative Commons licence and your intended use is not permitted by statutory regulation or exceeds the permitted use, you will need to obtain permission directly from the copyright holder. To view a copy of this licence, visit http://creativecommons.org/licenses/by/4.0.

Open access funding enabled and organized by Projekt DEAL.

Original article has been corrected.

